# Atomistic simulations shed new light on the activation mechanisms of RORγ and classify it as Type III nuclear hormone receptor regarding ligand-binding paths

**DOI:** 10.1038/s41598-019-52319-x

**Published:** 2019-11-21

**Authors:** Suwipa Saen-Oon, Estrella Lozoya, Victor Segarra, Victor Guallar, Robert Soliva

**Affiliations:** 1 Nostrum Biodiscovery, Jordi Girona 29, Nexus II D128, 08034 Barcelona, Spain; 2 Molecular Informatics Department, Almirall S.A., Laureà Miró 408-410, 08980 St. Feliu de Llobregat, Barcelona Spain; 3 Barcelona Supercomputing Center (BSC), Jordi Girona 29, 08034 Barcelona, Spain; 4 ICREA, Passeig Lluís Companys 23, 08010 Barcelona, Spain

**Keywords:** Computational biophysics, Mechanism of action, Computational chemistry

## Abstract

The molecular recognition of the RORγ nuclear hormone receptor (NHR) ligand-binding domain (LBD) has been extensively studied with numerous X-ray crystal structures. However, the picture afforded by these complexes is static and does not fully explain the functional behavior of the LBD. In particular, the apo structure of the LBD seems to be in a fully active state, with no obvious differences to the agonist-bound structure. Further, several atypical *in vivo* inverse agonists have surprisingly been found to co-crystallize with the LBD in agonist mode (with co-activator), leading to a disconnection between molecular recognition and functional activity. Moreover, the experimental structures give no clues on how RORγ LBD binders access the interior of the LBD. To address all these points, we probe here, with a variety of simulation techniques, the fine structural balance of the RORγ LBD in its apo vs. holo form, the differences in flexibility and stability of the LBD in complex with agonists vs. inverse agonists and how binders diffuse in and out of the LBD in unbiased simulations. Our data conclusively point to the stability afforded by the so-called “agonist lock” between H479 and Y502 and the precise location of Helix 12 (H12) for the competence of the LBD to bind co-activator proteins. We observe the “water trapping” mechanism suggested previously for the atypical inverse agonists and discover a different behavior for the latter when co-activator is present or absent, which might help explain their conflicting data. Additionally, we unveil the same entry/exit path for agonists and inverse agonist into and out of the LBD for RORγ, suggesting it belongs to the type III NHR sub-family.

## Introduction

**Figure 1:**
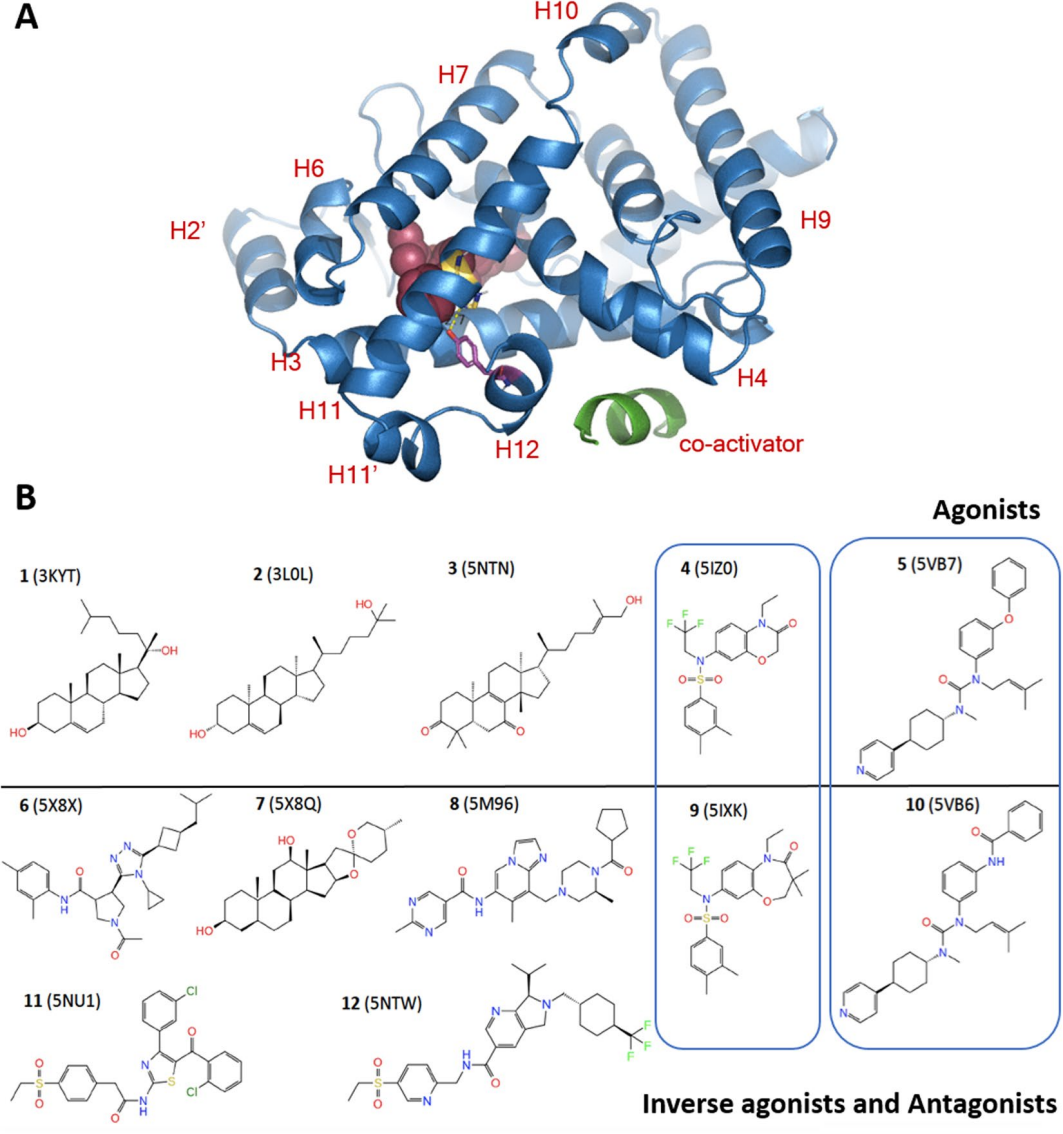
(**A**) Overall structure of the RORγ-LBD with compound **5** bound in the orthosteric site (represented as CPK, in purple). The most important α-helices are labeled in red, as is the agonist lock H-bond formed between H479 (H11, in yellow) and Y502 (H12, in pink), shaping the co-activator docking site. The co-activator protein is docked on the lower part of the LBD (colored green). (**B**) 2D structures of representative compounds with activity on RORγ. Agonists: compounds **1-5** on top; Inverse agonists and antagonists on the bottom: compounds **6-12**; **6-7** are found in complex with LBD and co-repressor. **8-9** are found in complex with LBD and neither co-activator nor co-repressor. **10-12** are found in complex with co-activator and feature water molecules in the interior of LBD next to H12; **10** breaks the agonist lock whereas **11-12** do not. The 2D structures are tagged with their respective Protein Data Bank entries, where each compound can be studied in complex with RORγ-LBD. The two MMPs simulated in this study are highlighted in the blue boxes: compounds **5** vs **10** and **4** vs **9**.

Retinoic-acid related-orphan-receptor-C (RORC or RORγ) belongs to the nuclear hormone receptor (NHR) superfamily. It plays a fundamental role in the maturation of IL-17 producing Th17 cells and thus is an attractive target for a variety of autoimmune diseases such as psoriasis and rheumatoid arthritis^1,2^. RORγ features a multidomain structure characterized by an N-terminal DNA binding domain (DBD) and a C-terminal ligand-binding domain (LBD) capable of binding ligands and recruiting co-activator or co-repressor proteins^3^. It is generally accepted that binding of agonists to the LBD leads to co-activator recruitment and gene transcription, whereas binding of inverse agonists leads to co-repressor recruitment and gene silencing. Additionally, some compounds behave as neutral antagonists, which may be defined as molecules that preclude binding of either co-activator or co-repressor proteins^4^. The functional outcome driven by these ligands is determined by a series of conformational transitions of the LBD. Numerous crystal structures have pointed to the position of Helix 12 (H12, also known as AF2) as the key structural element enabling or precluding co-activator or co-repressor binding. In particular, the position of H12 seems to be neatly controlled by a key hydrogen bond between Y502 (located at H12) and H479 (located at H11), which has been termed “agonist lock”^5^ (see Fig. 1A). This single H-bond and the contact of this H479-Y502 duplet with neighboring F506 seems to properly position H12 for co-activator binding. On the other hand, its disruption leads to displacement or unfolding of H12 and either co-repressor binding (inverse agonists) or no protein binding (antagonists).

Remarkably, many bibliographic references show that very subtle variations in the structure of the different RORγ ligands can have a huge impact on their functional outcome. Thus, there are several matched molecular pairs (MMP)^6,7^ of active ligands where subtle changes lead to dramatic shifts from potent agonism to potent inverse agonism. Figure 1B displays a varied set of RORγ-LBD ligands where agonists and inverse agonists are represented^4–9^. Examples of MMPs of highly similar ligands with opposed functional outcomes can be found (see compounds **4** vs. **9** or **5** vs. **10**)^6,7^. Generally, it is accepted that ligands behaving as agonists must somehow stabilize the agonist lock and H12, whereas ligands behaving as antagonists and inverse agonists disrupt this interaction by directly H-bonding to H479, pushing H11 or disrupting the contact interface between helices H11, H11’ and H12.

In spite of this simple theoretical description, the actual recognition landscape of the RORγ-LBD seems to be more complex than initially anticipated, probably involving dynamic aspects associated to the conformational transitions and the role of solvent that are not completely captured by experimental techniques. Firstly, the apo form of the LBD solved by different groups seems to be in a fully active state, with agonist lock perfectly formed and H12 correctly positioned, allowing proper docking of co-activator proteins^4,7^ (PDB IDs: 5  x8w, 5  x8u 5vb3). In other words, apparently there are no structural differences between apo and agonist-bound crystal structures.

Secondly, some ligands proven to be inverse agonists by a variety of *in vitro* and *in vivo* experiments have been surprisingly found to co-crystallize with the LBD in a fully active state, i.e. in complex with co-activator protein^5–7,10^ (see Fig. 1B for a few cases). Intriguingly, although they allow agonist-position of H12 and recruitment of co-activator, some of them disrupt the agonist lock (for instance compound **10** disclosed by Boehringer, which H-bonds to H479, Fig. 1B) while others do not (Fig. 1B, compounds **11** and **12**). A common theme for this type of puzzling inverse agonists is the presence of water molecules inside the LBD in the vicinity of the H11-H12 interface, together with the bound ligand. This has prompted to propose a “water trapping” mechanism^5^, whereby these atypical inverse agonists behave as agonists in the crystal structures but might be unstable *in vivo* due to the destabilizing effect of trapped solvent molecules. In view of this phenomenon, caution is advised, as access to a crystal structure where a novel ligand-LBD pair is in complex with co-activator does not directly equate to *in vivo* agonism.

Thirdly, the LBD is compact globular structure rich in α-helices (Fig. 1A). Its ligand-binding site is located deeply at the core of the domain. Inspection of all co-crystal structures does not reveal an obvious entry path for this deep buried site. Although there are a series of theoretical studies on how ligands access the LBD for several NHRs modulators such as PPARγ^11^, mineralocorticoid receptor and glucocorticoid receptor^12,13^, no information exists on how ligands diffuse in or out of the RORγ-LBD. Very recently, a review by Fisher and Smiesko has been published classifying all NHRs in different families (Types I-VI) based on the pathways that ligands travel during access to, or egress from the LBD^14^. It further discusses that generally, the majority of computational studies suggest no major re-arrangements amongst the helices are needed to enable binding and argue that some works propose binding and unbinding pathways to be the same. The review further notes that most of the published studies concentrate on either access or exit paths, but very rarely on both for a given NHR. Thus, a complete study of both agonists and inverse agonists accessing and exiting the same NHR is lacking.

The incomplete understanding of the structural transitions taking place at the RORγ-LBD prompted us to launch a series of atomistic simulations in different scenarios with the aim of elucidating the role played by the different types of ligands, the solvent and the interplay between the different structural elements composing the LBD. Compounds **5** and **10** (Fig. 1B) disclosed by Boehringer^7^ and **4** and **9** from Biogen^6^ were taken as a prototypical first test set for studying RORγ-LBD, as they represent MMPs where slight structural variations result in changes from agonism to inverse agonism, and because they have been studied by both X-ray crystallography and NMR. Our simulations, by representing a fully flexible system in a solvated environment, captured relevant details not readily derivable by experimental techniques. A comparison between apo vs. ligand-bound states uncovered differences in the structural stability of the agonist lock and the interaction energies between H12, co-activator and the rest of the LBD. They gave clues on the hydration of the LBD for the atypical inverse agonists and suggested a possible explanation for their disconnection between X-ray information and *in vivo* pharmacology, and uncovered a common entry/exit path for all types of ligands that seems to be alternative to many other nuclear hormone receptors.

## Results and Discussion

A series of systems were set up and simulated by unbiased molecular dynamics (MD) simulations. As a representative set of RORγ in different contexts, we took the work recently published by Boehringer, where the LDB was solved by X-ray crystallography^7^ in the following scenarios: i) the apo form (pdb entry 5vb3), termed “apo” throughout; ii) agonist-bound form, LBD in complex with compound **5** (pdb entry 5vb7), termed “ago” throughout; iii) inverse agonist-bound form, LBD in complex with compound **10** (pdb entry 5vb6) - the latter was started with and without the trapped water next to compound **10**, which we termed “iag” and “iag-nowater” respectively, with the goal of testing the water trapping hypothesis. Additionally, the “ago” and “iag” complexes where also simulated in absence of co-activator and termed “ago-noco” and “iag-noco” respectively, to check for the stability induced by these two compounds on the docking site of co-activator proteins when the latter are absent. This gave a total of 6 different MD simulation systems. Furthermore, the full binding and unbinding event for compounds **5** and **10** were explored by exhaustive-sampling PELE (Protein Energy Landscape Exploration) simulations in several conditions.

Additionally, a second MMP published by Biogen^6^, with agonist compound **4** and inverse agonist compound **9** were included in the study to compare the effects agonist/inverse agonist have on the conformational rearrangements influencing the co-activator docking site. Simulation of the agonist complex (compound **4**) was started from its crystal structure (pdb entry 5iz0^6^) in absence of co-activator and termed “4ago-noco”. Co-crystal structures of inverse agonist **9** revealed unfolding of the H11 and H12 helices (pdb entry 5ixk^6^) Thus, in order to study the dynamic disruption of the docking site for compound **9**, its simulation was started by docking and minimizing it on the RORγ-LBD as bound to compound **4** (5iz0), the latter system being termed “9iag-noco”.

### Dynamic behavior of the apo vs. agonist-bound RORγ LBD

**Figure 2:**
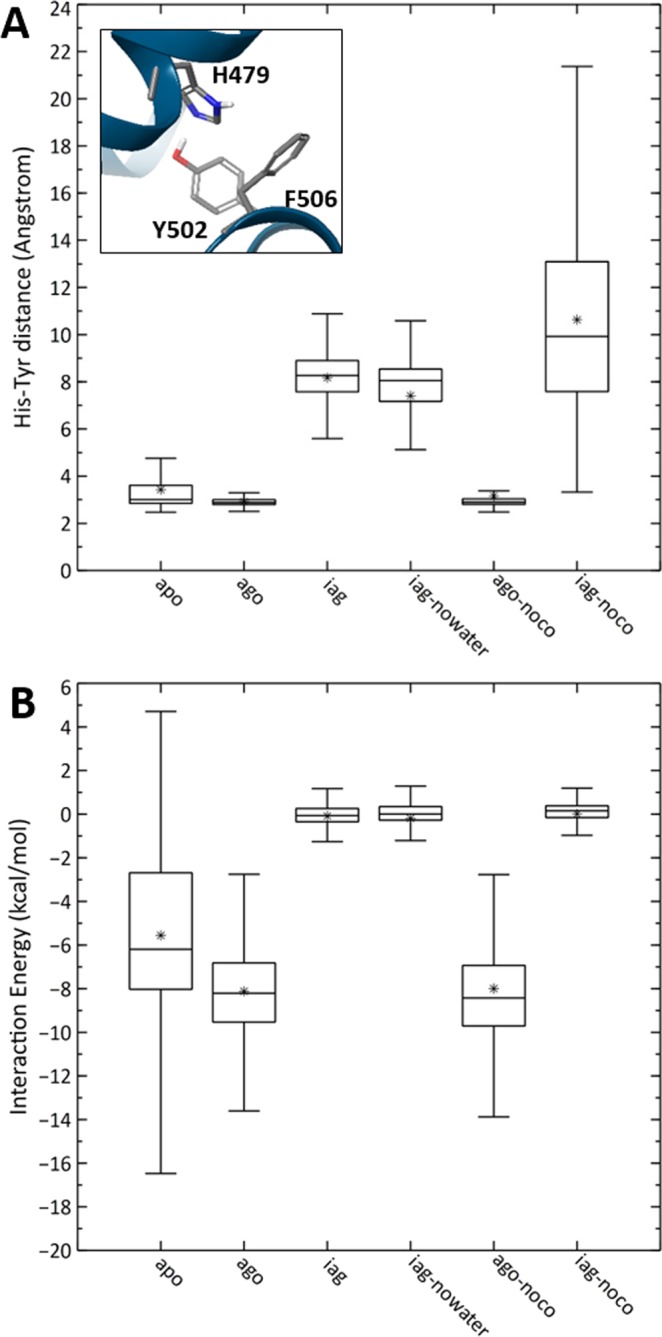
(**A**) Box plots of H479:Nε-Y502:OH distance (Angstroms) along the MD trajectories for each of the 6 simulated systems of the Boehringer MMP; (**B**) Box plots of the interaction energies (kcal/mol) for the H479-Y502-F506 triplet (see inset figure in A) for each simulated system. Box plots are generated from values obtained from four independent 0.5μs trajectories for each system where the whisker-bar represents the range of minimum and maximum values excluding outliers, the median is represented by a line subdividing the box and the mean is represented by asterisk (*). (apo, ago, iag, iag-nowater, apo-noco, iag-noco correspond to simulations of apo structure of RORγ-LBD, RORγ-LBD bound to compound **5**, RORγ-LBD bound to compound **10**, RORγ-LBD bound to compound **10** starting with no water in the orthosteric site, RORγ-LBD bound to compound **5** in absence of co-activator and RORγ-LBD bound to compound **10** in absence of co-activator, respectively).

**Figure 3:**
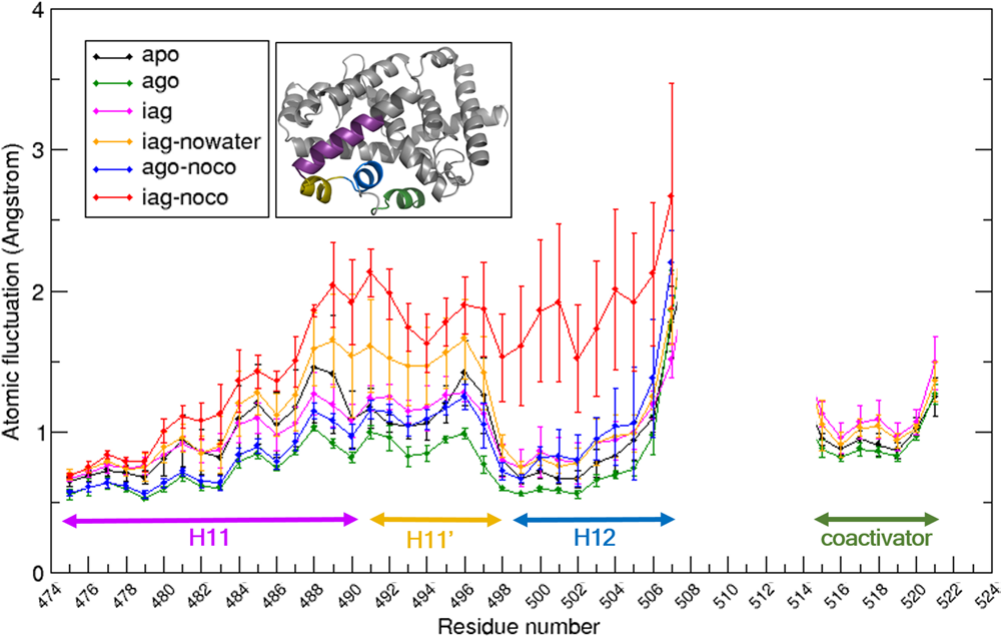
Atomic fluctuation (Y-axis in Angstrom) for all backbone atoms in the different simulated complexes, each represented with a different color-coding (see first inset). The X-axis represents amino acid numbers, with the different secondary structure segments involved in the structural transitions (H11, H11’, H12, co-activator) marked in different colors (the color correspondence can be seen in the second inset). Values obtained from averaging over four independent 0.5μs trajectories for each system along with standard deviation value. The plot excluded the highly mobile part contributed from the loop residue 508 to 514 linking H12 to co-activator and C-terminal residues.

We first turned our attention to the apo form of RORγ-LBD. An analysis of several apo crystal structures available (for instance 5 x8w^4^ and 5vb3^7^) reveal that apo LBD is in its active state, with a stably formed agonist lock (distances ca. 3 Å between H479 Nε and Y502 OH), agonist-positioned H12 and docked co-activator proteins. The differences between apo and agonist-bound could be driven either by dynamic aspects or they might originate due to crystal packing effects deviating the conformations from those found in solution. To check this, we compared the four apo simulations starting from 5vb3 vs. the four independent agonist LBD-compound **5** simulations starting from 5vb7. As shown in Fig. 2A (first two columns on the left and also in Supplementary Information (SI) Figs. S1 and S2), although the key H479-Y502 H-bond median distance is nearly identical for apo vs. agonist-bound (ca. 3 Å), the degree of fluctuation is much higher for the former than for the latter. In fact, the apo trajectories reveal 12.52% of the time the agonist lock is broken (>4 Å), something not captured by the X-ray structures. In contrast, it is only 0.08% found broken for the agonist-bound simulations. Boehringer scientists performed a series of *ab initio* calculations of the H479-Y502-F506 triplet and concluded the interaction energy for this triplet is very strong for the apo RORγ-LBD, a key structural feature not seen with other NHRs such as PPARγ. An analysis of our trajectories shows, indeed, strong median interaction energy of ca. −6.19 ± 3.25 kcal/mol (Fig. 2B) for apo. However, our simulations point to an enhanced triplet interaction for the agonist **5**-LBD complex, giving an average of −8.21 ± 1.98 kcal/mol, i.e. 2 kcal/mol stronger than the apo. Moreover, the interaction energy for the triplet has a much narrower distribution in the agonist-bound trajectory. In other words, agonist **5**-LBD is not only energetically more favorable in the active conformation than apo, it is also more stable revealing a much lower structural fluctuation. This can also be seen in Fig. 3, where the atomic fluctuation for all backbone atoms is visualized as an average value extracted from the four independent MD simulations for each residue. Figure 3 clearly reveals that all amino acids located in H11, H11’, H12 and co-activator fluctuate much more for apo (black line) than for ago (green line). These fluctuation differences might explain the enhanced gene transcription capability of RORγ when in complex with full agonists vs. apo.

### Dynamic behavior of the agonist vs. inverse agonist bound LBD - testing the “water trapping” hypothesis

Analysis of the two trajectories for the **5**-LBD and **10**-LBD complexes allowed a direct comparison between a closely related MMP with disparate functional activity. Because compound **10** breaks the agonist lock by directly H-bonding to H479, the H479-Y502 distance is around 7 Å in the crystal structure. Its distribution seen over the four trajectories is close to this value, centered around 8 Å (Fig. 2A), but the degree of fluctuation is high, as the H479-Y502 distance ranges from 6 to 11 Å. In line with the *ab initio* calculation on the X-ray structure performed by Boehringer^7^, the H479-Y502-F506 interaction energy for the **10**-LBD complex is much lower (median of ca. −0.06 ± 0.65 kcal/mol) than for the **5**-LBD complex (−8.21 ± 1.98 kcal/mol) or even the apo 6.19 ± 3.25 kcal/mol). Thus, the presence of compound **10** in the orthosteric site, although still compatible with co-activator binding, leads to a destabilization of the H12 helix even when compared to the apo structure.

An interesting feature of the puzzling inverse agonists such as compound **10** is the presence of a water molecule “trapped” with the ligand at the H11-H12 interface. In order to test the “water trapping” hypothesis, we run four simulations of the **10**-LBD complex starting with and without the single water molecule found in its crystal structure. The purpose of the four “dehydrated” trajectories was to check whether the whole system would be stable in absence of this critical solvent molecule. The simulations with and without this water molecule turned out to be nearly identical in terms of H479-Y502 distance and triplet interaction (see Fig. 2A,B). Interestingly, in the “dehydrated” **10**-LBD complex, water molecules started diffusing back inside the ligand binding site early-on in the simulations (before 20 ns in three independent trajectories, but only in one trajectory at 90 ns – see SI Fig. S3). Thus, the “dehydrated” versions of bound compound **10** seem to be stable and lead to a quick water refilling of the binding site, confirming the water trapping process.

**Figure 4:**
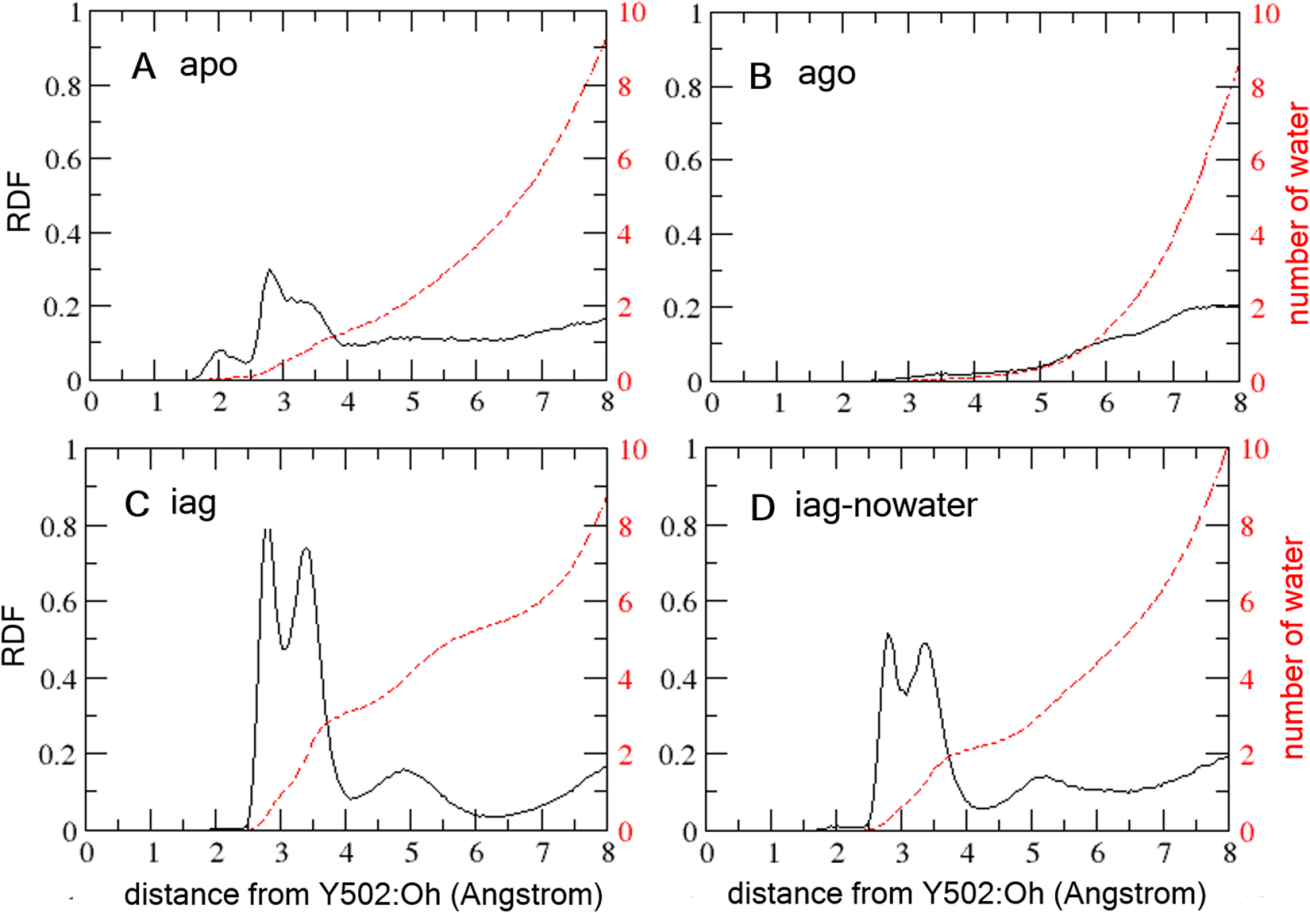
Radial distribution functions for the solvation around the H479-Y502 agonist lock centered on the OH atom of Y502 for (**A**) the apo, (**B**) **5**-LBD, (**C**) **10**-LBD and (**D**) **10**-LBD started without any water molecules inside the binding site. The black and red profiles show the water density and aggregate number of water molecules along the radius, respectively. Only results of the first MD simulation are shown for each system.

We carried out an exhaustive analysis of the hydration around the agonist lock for the all the simulated systems. Figure 4 reveals marked differences among them in the radial distribution function (RDF) around OH atom of Y502. The less hydrated system in the vicinity of the H11-H12 interface is the agonist bound LBD, as the first water molecule is integrated in the 6 Å radius. The second less hydrated is the apo system, where the first water molecule is integrated in a ca. 3.5 Å radius. The systems with the inverse agonist are by far the most hydrated in the vicinity of Y502, showing two water peaks around 2.5 and 3.5 Å. These trajectories reveal 2–3 water molecules in the H11-H12 interface for the **10**-LBD-co-activator complex.

Thus, our simulations suggest compound **10** seems to be compatible with co-activator only by recruiting water molecules inside the binding cavity, and the degree of hydration of the H11-H12 contact area is much higher than the apo form, a phenomenon that could also contribute to its high degree of fluctuation and therefore its lack of stability *in vivo*.

### Conformational stability of agonist-LBD and inverse agonist-LBD complexes in absence of co-activator

Multiple atypical inverse agonists have been co-crystallized or even soaked in different conditions together with a range of different co-activator proteins^4–8^, always giving the same disconnection between structure and pharmacological outcome. It was decided to also simulate **5**-LBD and **10**-LBD in absence of co-activator peptide, which was removed from their respective X-rays. This would allow us to study the dynamic behavior of systems that seem to be inaccessible via X-ray crystallography.

The results are also found in Fig. 2 (two rightmost columns). The median distance for the H479-Y502 agonist lock in the four MD trajectories of agonist **5**-LBD complex *without* co-activator is around 3 Å, and the interaction energy of the H479-Y502-F506 triplet is around −8.50 ± 2.25 kcal/mol, mirroring the behavior seen when co-activator is present. In other words, the agonist **5**-LBD complex with and without co-activator is nearly identical in terms of both structure and flexibility. However, we found striking differences when comparing the compound **10**-LBD complex with and without co-activator. When co-activator is present, although the fluctuation and solvation are increased as discussed above, the structure is kept close to the X-ray. However, in absence of co-activator, the **10**-LBD complex clearly deviates from the structure seen in the X-ray. This is also seen in Fig. 2, for both H479-Y502 distance and H479-Y502-F506 triplet interaction energy. Additionally, the atomic fluctuation of amino acids located in H11, H11’, H12 clearly reveals the largest mobility amongst all the simulated systems (see the red line in Fig. 3).

**Figure 5:**
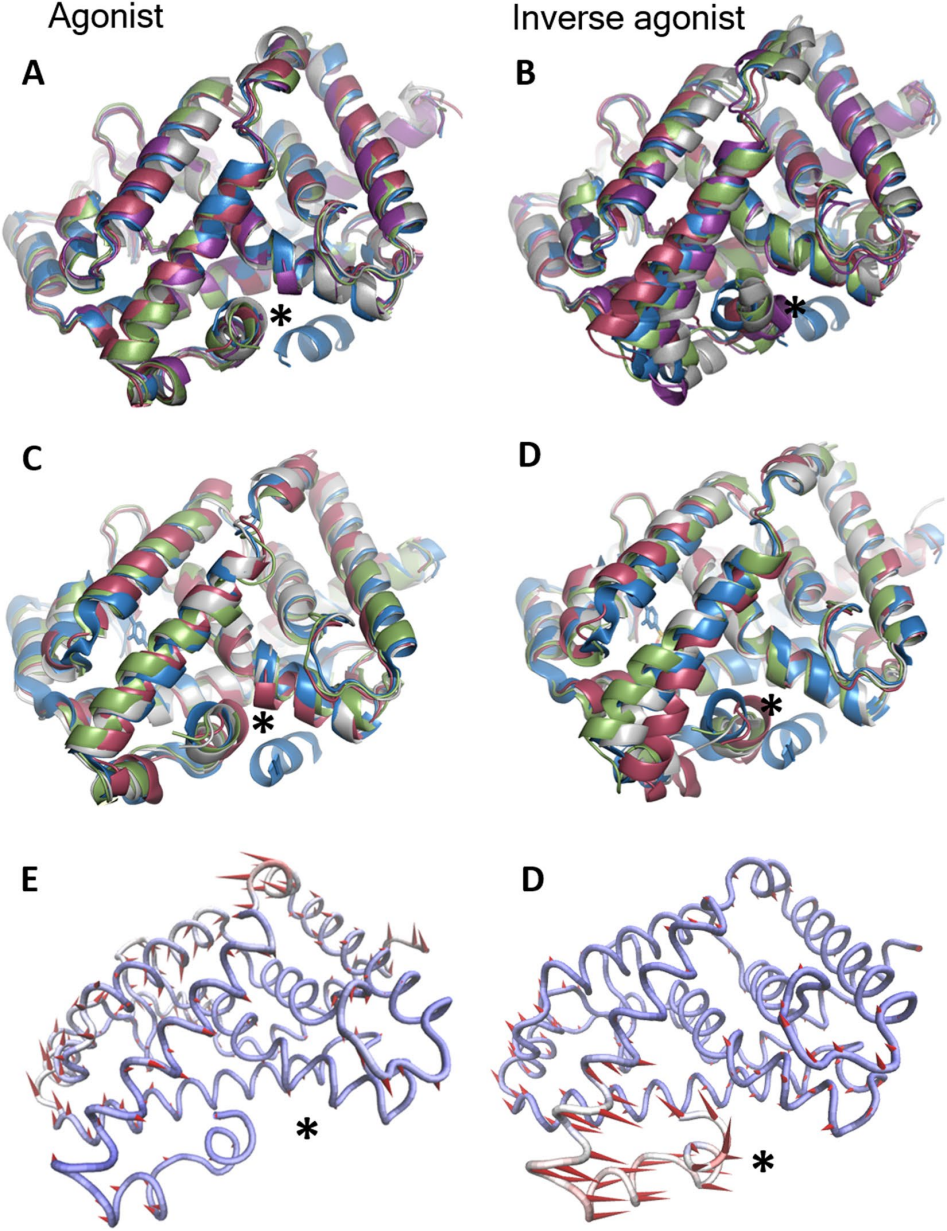
The RORγ-LBD-co-activator complex final structure for one of the apo simulations is represented in blue (the co-activator is properly docked thanks to the correct positioning of H12). The simulations of agonist compound (**A**) **5**-LBD and (**C**) **4**-LBD complex in absence of co-activator still show a correctly positioned H12. However, inverse agonist compound (**B**) **10**-LBD and (**D**) **9**-LBD complex in absence of co-activator leads to a conformational transition, where H12 invades the docking site for co-activator, precluding its binding (marked with an asterisk). For both MMP, this is seen for all 4 independent simulations of the same system in the absence of co-activator represented in gray, red, green and magenta color, respectively. Dynamic motion capturing helix H12 mobility is exemplified by the first Principal Component for the system in absence of co-activator for (**E**) agonist **5-**LBD and (**F**) inverse agonist **10-**LBD.

**Figure 6:**
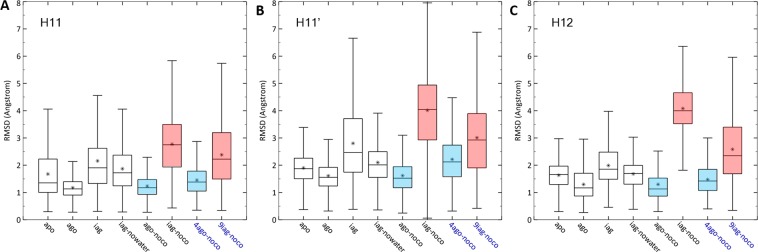
Box plots of Root-mean-square-deviation (RMSd) for backbone atoms (Y-axis in Angstrom) for helices (**A**) H11 (**B**) H11’ and (**C**) H12. In both MMPs (Boehringer’s and Biogen’s), a significantly higher mobility is seen in inverse agonist bound systems vs. agonist bound systems in absence of co-activator (red and blue respectively). Values obtained from 4 independent 0.5μs and 3 independent 0.6μs trajectories for Boehringer and Biogen systems, respectively. The whisker-bars represent the range of minimum and maximum values excluding outliers, the “median” is represented by a line subdividing the box and the “mean” is represented by asterisk (*).

Although the sequence of events involved in the formation of a fully functional transcriptional complex *in vivo* is not known, it can be derived from our results that a RORγ-LBD which first binds agonist compounds **4** and **5** in solution will still be able to bind co-activator proteins, as the co-activator docking site is preserved (see Fig. 5A,C). However, our simulations suggest that a RORγ-LBD which first binds inverse agonists such as **9** or **10** will undergo a transition to a structure for which H11, H11’ and H12 would be wrongly positioned for co-activator binding, as it partly invades the docking site (see Fig. 5B,D). This is also captured by the PCA analyses performed on the trajectories without co-activator. The first principal component for the compound **5**-LBD does not involve the deformation of the docking site, whereas the principal component of compound **10**-LBD changes the shape where co-activator proteins fit, with H12 partly invading it (Fig. 5E vs F). These observations are further supported by quantitative analyses of the RMSd for helices H11, H11’ and H12 for agonist-bound vs. inverse agonist bound simulations in absence of co-activator. A higher degree of displacement is highlighted for the latter (see Fig. 6).

### Entry/exit paths for RORγ LBD binders

An interesting and unresolved question remains related to how RORγ-LBD active compounds access the binding site, which is at the core of the LBD. The binding process for NHR ligands has been studied by simulation^12^. A recent review summarizes what is known on (un)binding paths for the whole NHR family based on multiple studies mostly employing different enhanced sampling MD techniques^14^. This study classifies the whole family into Types I-VI based on the spaces between the LBD helices the ligands traverse on their access/egress paths. Notably, the entry paths for androgen receptor (AR), estrogen receptor (ER), glucocorticoid receptor (GR), mineralocorticoid receptor (MR) and progesterone receptor (PR) with their respective endogenous ligands have recently been studied with multiple Monte Carlo simulations with the PELE platform^12,13^, concluding that simulations suggest all of them belong to Type II (the main entry path is a narrow hole opened up between helices H3, H7 and H11 (identified in Fig. 1A)). We performed the same type of Monte Carlo simulations with compounds **5** and **10** on the RORγ-LBD.

**Figure 7:**
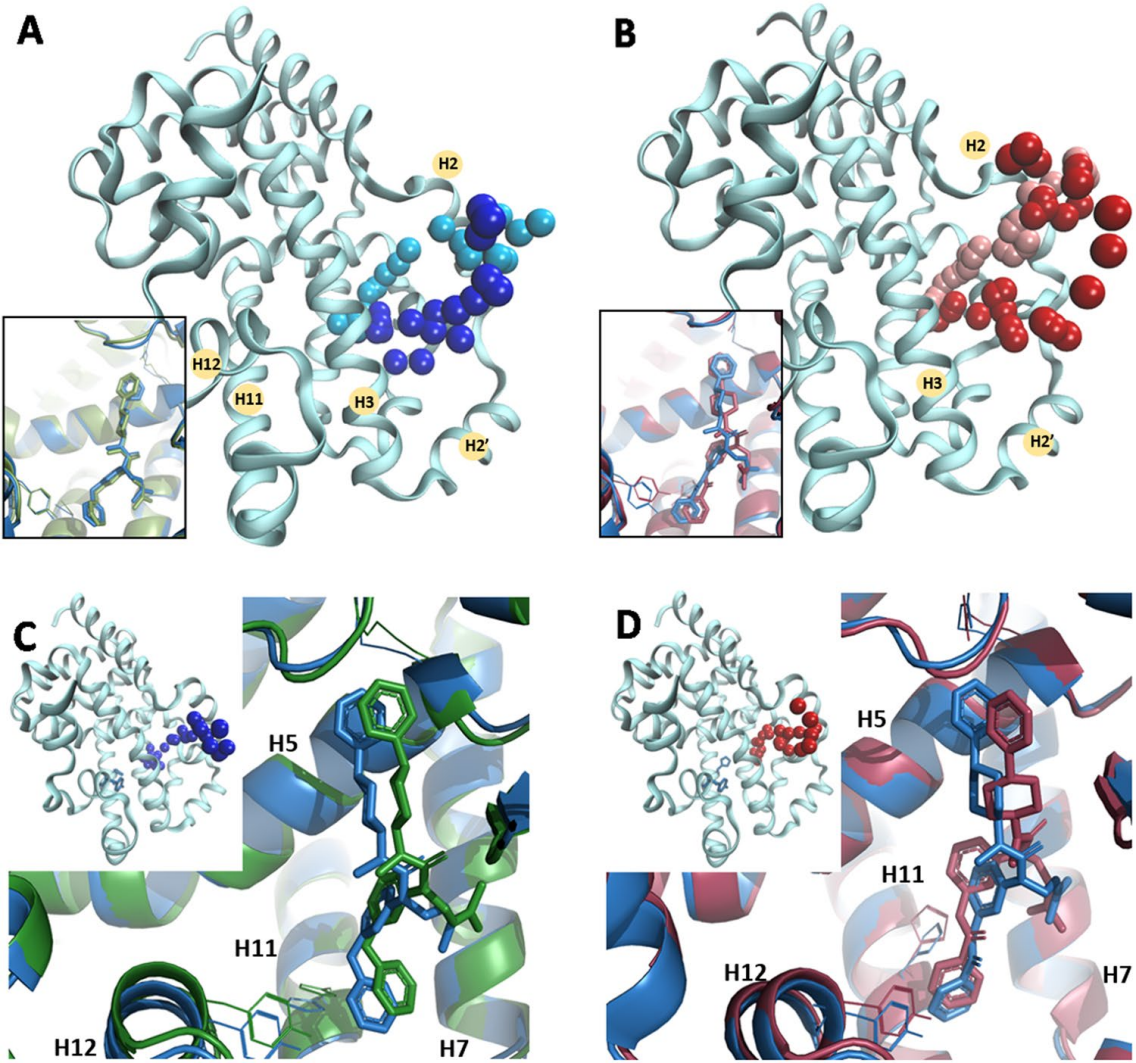
PELE simulations illustrate the ligand exit/entry pathway for (**A**) agonist (light and dark blue color, respectively) and (**B**) inverse agonist (light and dark red color, respectively), against their respective crystal structures. The spheres represent the ligand center-of-mass position in the trajectories. The insets show superposition of the best-docked pose of agonist (green) and inverse agonist (red) to their crystal structures (blue) with RMSd of 1.02 Å and 1.52 Å, respectively. PELE entry path simulations for (**C**) agonist compound **5** and (**D**) inverse agonist compound **10** against the apo protein. Superposition of the best pose from the PELE entry simulations for **5** and **10** to their crystal structure (blue color) with RMSd of 1.73 and 2.31 Å, respectively. The entry pathway (sphere is ligand’s center-of-mass) is illustrated in the inset.

We first run a series of simulations with both compounds bound as they are found in their respective crystal structures, in search for their exit paths out of the LBD. The only exit path found was through a hole between H2, H3 and the beta-hairpin, which we termed “backdoor” (see Fig. 7A,B), away from the H3/H7/H11 tunnel mentioned above composing the entrance for many other NHR. Remarkably, this was seen for both compounds, agonist **5** and inverse agonist **10**.

For the entry path, bearing in mind what had been found the other NHRs, we first placed agonist **5** in the vicinity of the H3/H7/H11 interface. Surprisingly, the only way for compound **5** to diffuse inside the LBD was again through the backdoor path (Fig. 7A), although all the copies were starting remotely from it. That is, not one single copy found its way through the entrance path found previously for ER, AR, PR or GR based on PELE trajectories. Simulations were very successful as the final pose for **5** was only at a remarkable 1.02 Å RMSd with respect to its X-ray pose (Fig. 7A, inset). We carried out the same calculation for inverse agonist **10** (against its crystal structure). Again, this compound diffused inside of the LBD only through the backdoor. The RMSd of the final pose generated for **10** was only 1.52 Å with respect to the experimental pose and fine details were retrieved such as the H-bond to H479 via its amide NH group (Fig. 7B). A careful study of the interactions taking place between **5** or **10** and the residues lining the backdoor seemed to point to hydrophobic interactions as the main driving force behind the initial phases of binding. In particular, the movement of the beta hairpin connecting H5 and H6 (N to C terminal sequence: VFFEGK) with respect to the central portion of H3 (N to C terminal sequence: CAHHLTEA) seemed to be key to further open up the hole (pocket/cavity/entrance) in a breathing motion for both compounds to sneak inside the cavity.

**Figure 8:**
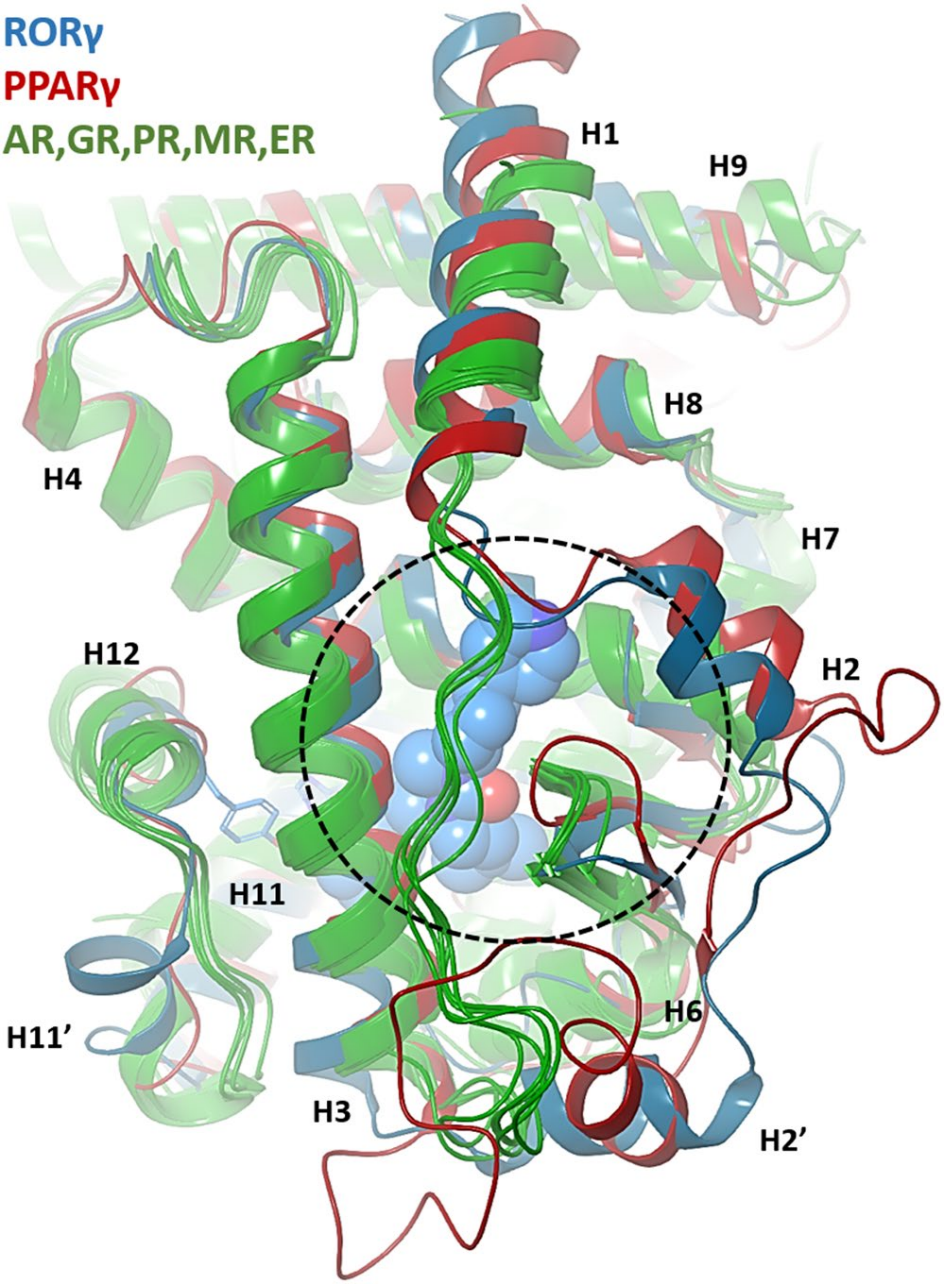
Structural alignment of the nuclear hormone receptor family from the following x-ray structure PDB IDs: RORγ (5vb7); PPARγ (1prg); AR (2q7j); ERα (1qkt); GR (4p6x); MR (2aa2) and PR (1a28). Coloring for RORγ and PPARγ are in blue and red, respectively while the others are in green. The agonist molecule and the H479-Y502 “agonist lock” in RORγ are shown with sphere and stick representation, respectively. The area depicted in dash-circle illustrates a “backdoor” entry in RORγ and PPARγ while it is totally covered with a long loop across the entrance in other NHRs proteins.

Finally, the same entry path simulations were repeated, now against the apo form of the LBD, with the purpose of ruling out any possible bias the use of the holo structures might cause. Consistently, compounds **5** and **10** diffused inside the orthosteric site of the apo structure through the backdoor and ended up at a remarkable RMSd of 1.73 Å and 2.31 Å of their corresponding X-ray structures, respectively. Once dynamically docked, compound **5** is still compatible with a stable agonist lock, whereas compound **10** positions its phenyl ring in between H479 and Y502, effectively breaking it (Fig. 7C,D). In view of the classification proposed by Fisher and Smiesko^14^, RORγ is then a type III NHR. Other members of this class are believed to be FXR, RARγ and PPARγ. On comparing the structures of RORγ and PPARγ on the one hand and ER, AR, GR, PR and MR on the other, one striking structural difference became obvious. The “backdoor” hole (cavity/entrance) discovered by our unbiased simulations (and for PPARγ) is blocked in the second group, where there is a long segment connecting H1 and H3 hindering access to the core of the LBD, see Fig. 8. Thus, our simulations strongly support the binding of ligands for RORγ-LBD is type III, involving the backdoor path and clearly differentiates it from that of Type II members such as AR, MR or GR. Finally, this finding is further supported by very recent publications by AstraZeneca on the development of potent RORγ 4-aryl-thienylacetamide inverse agonists^15^. Their previous fragment-based efforts^16^ discovered DMSO molecules binding on the rim of the “backdoor”. By elaborate medicinal chemistry, they could develop derivatives that bind in the interior of the LBD and extend to the surface via the backdoor, effectively mimicking the sub-pocket occupied by the DMSO molecules, which hydrogen-bond to E379, right at the middle of the VFFEGK sequence noted above.

## Conclusion

The atomistic simulations presented here complement what is known on the activation/inactivation mechanisms of RORγ from crystallographic studies. We reveal the RORγ apo structure, although seemingly in the fully active state, features a H11/H11’/H12/co-activator area that fluctuates much more than an agonist-bound counterpart, leading to intermittent agonist lock breakage in solution. Our simulations also demonstrate that the atypical inverse agonists do indeed trap water molecules in the vicinity of the H11/H12 interface, even in cases where the initial simulated structure lack such water molecules, as they rapidly diffuse back inside the core of the LBD. Moreover, they also reveal that these atypical inverse agonists, although seemingly compatible with an active state, lead to a very high degree of structural destabilization, which becomes even more pronounced when the inverse agonist-RORγ-LBD complex is formed in *absence* of co-activator. Finally, our simulations for entry and exit paths for agonists and inverse agonists suggest RORγ is a type III NHR (entry/exit path composed of H2, H3 and the beta-hairpin connecting H5 to H6 for RORγ-LBD) which coincides with PPARγ, and is opposed to what is found for other members of the NHR family such as ER, AR, GR or MR.

## Methods

### System preparation

All structures were first pre-processed and refined with Protein Preparation Wizard^17^ of Maestro. This pipeline conveniently corrects for deficiencies such as missing side chains and loops, adds hydrogen atoms, checks for protonation states of ionizable amino acids (at physiological pH) and flips wrongly assigned Asn and Gln side chains. The resulting structures were visually inspected for final quality control.

### Molecular dynamics simulations

MD simulations were carried out with NAMD 2.12^18^ with the AMBER ff99sb force field^19^. Explicitly solvated (TIP3P waters^20^) systems were simulated in the NPT ensemble in a truncated octahedron boxes of sizes 90 Å × 90 Å × 90 Å. The systems were brought to neutrality with the addition of sodium and chlorine ions, which were added to simulate physiological ionic strength. Atomic charges for ligands (compounds **5,10, 4 and 9)** were obtained by the RESP methodology^21^, calculated at HF/6-31 G** level of theory with the Jaguar program^22^ together with GAFF parameters^23^ afforded by the AmberTools.

In order to increase the statistics of all parameters studied, four independent 0.5μs simulations were carried out for each of the 6 systems from Boehringer compounds **5** and **10**, giving a total of 24 trajectories. For Biogen compounds **4** and **9**, due to the significant structural disruption observed in crystals complexes arose from subtle change in chemical structure (see Fig. 1 compound **4** and **9**), we performed 0.6μs of three independent simulations. That is, we accumulated ~ 2μs for each system under study coming from aggregating three to four independent runs. The 8 systems were first equilibrated by applying the following multi-step protocol. Each system was minimized using default minimizer in NAMD; a conjugate gradient and line search algorithm, with all heavy protein atoms restrained at their initial position applying restraining force constant of 10 kcal/mol.Å^2^. Initial velocity was assigned randomly through the different seed numbers for each system during gradually heating process from 0 to 298 K over the first 150 ps with harmonic constraints assigned to the protein-ligand complex. The protein was continuously equilibrated over 50 ns by gradually reducing the harmonic constraint on the heavy atoms from 50 to 0 kcal/mol.Å^2^, while weakly restraining the ligand to its initial conformation. Then, the harmonic restraint on ligand was slowly released during 50-100 ns, to fully equilibrate the protein-ligand complex. Overall, the whole of the equilibration phase takes 15 separate steps and a total of 100 ns in length. SHAKE^24^ was used to constrain chemical bonds, which allowed to use an integration step of 2 fs. Periodic Boundary Conditions and the Particle Mesh Ewald methods were used to treat long-range electrostatic effects. In the simulation run, snapshots were saved every 10 ps. All the analyses for distances, RMS atomic fluctuation, linear response interaction energies (LIE) and Principal Component Analysis (PCA)^25^ were carried out with CPPTRAJ^26^. The VMD-integrated Normal Mode Wizard (NMW) was used to construct porcupine plots^27^.

### PELE simulations

PELE (Protein Energy Landscape Exploration) is a highly efficient Monte Carlo (MC) algorithm^28^ that has been extensively applied to a variety of molecular recognition problems^12,29,30^. The basic algorithm works as follows: each MC move consists of three main steps: i) ligand and protein perturbation; ii) side chain rotamer sampling; and iii) system minimization. The ligand is perturbed in a series of rotations and translations, whereas the protein is perturbed based on a minimization with constrained displacements along the Cα-atoms following a set of given modes which can derived from an anisotropic network model (ANM)^25,31^ or PCA^29^. The resulting structure obtained in the final minimization step is accepted or rejected by applying a Metropolis criterion. The all-atom force field used is OPLS-2005^32^. The solvent is implicitly represented by the addition of the continuum solvation model by Onufriev-Bashford-Case^33^. This algorithm has been shown to perform extremely well at reproducing experimental binding modes in cross-docking scenarios, and in non-biased exploration of ligand (entry and exit) migration pathways, including applications to NHR^12^.

Entry and exit simulations for compounds **5** and **10** were run on 216 cores, typically lasting 10–15 hours each on a compute cluster using Intel Xeon Gold 6140 CPU processors. Exit simulations used the protein-ligand bound complexes (pdb entry 5vb7 and 5vb6 for compounds **5** and **10**, respectively) as a starting point. The ligand was perturbed with random translations (from 0.50 and 1.00 Å) and rotations (0.05 and 0.15 radian) using PELE’s adaptive procedure to achieve a non-biased continuous exit pathway^29^. For the entry simulations, we first began with the free search pathway by placing the ligand in four different positions at the bulk solvent and using the sampling sphere of 50 Å from the center of the active site. Similar parameters to the exit simulations were used, but with larger translations (from 1.25 and 2.50 Å) during ligand perturbations. Ligand’s surface area (SASA) was used to enhance the ligand sampling by increasing the translation to 5.00 Å when 85% exposed to the solvent, and decreasing to 0.75 Å when buried inside the protein (15% exposed). The direction of such perturbations was kept for two consecutive steps. The newly developed adaptive-PELE scheme was also used^29^. After a short simulation of 10 Monte Carlo steps, the adaptive scheme clustered the ligand position and new initial conditions were chosen, prioritizing those clusters with less population. Later, PELE entry simulations by using restricted sampling sphere of 15 Å, from the center of exiting area were carried out for the migration of compounds **5** and **10** to the apo protein (pdb entry: 5vb3) by applying the same adaptive procedure and perturbation parameters.

## Supplementary information

Atomistic simulations shed new light on the activation mechanisms of RORγ and classify it as Type III nuclear hormone receptor regarding ligand-binding paths
