# Supplementary material for: Atomistic simulations shed new light on the activation mechanisms of RORγ and classify it as Type III nuclear hormone receptor regarding ligand-binding paths
Source: Sci Rep. 2019 Nov 21;9:17249. doi: 10.1038/s41598-019-52319-x (PMC6872664; doi:10.1038/s41598-019-52319-x)
Supplement: Supplementary file 1 — Atomistic simulations shed new light on the activation mechanisms of RORγ and classify it as Type III nuclear hormone receptor regarding ligand-binding paths [file 41598_2019_52319_MOESM1_ESM.pdf]

Atomistic simulations shed new light on the activation mechanisms of RORγ and classify it as Type III nuclear hormone receptor regarding ligand-binding paths

Suwipa Saen-Oon[1], Estrella Lozoya[2], Victor Segarra[2], Victor Guallar[3,4] & Robert Soliva[1,*]

[1]Nostrum Biodiscovery, Jordi Girona 29, Nexus II D128, 08034, Barcelona, Spain

[2]Molecular Informatics Department, Almirall S.A., Laureà Miró 408-410, 08980, St. Feliu de Llobregat, Barcelona, Spain

[3]Barcelona Supercomputing Center (BSC), Jordi Girona 29, 08034, Barcelona, Spain

[4]ICREA, Passeig Lluís Companys 23, 08010, Barcelona, Spain

[*]rsoliva@nostrumbiodiscovery.com

Supplementary Information

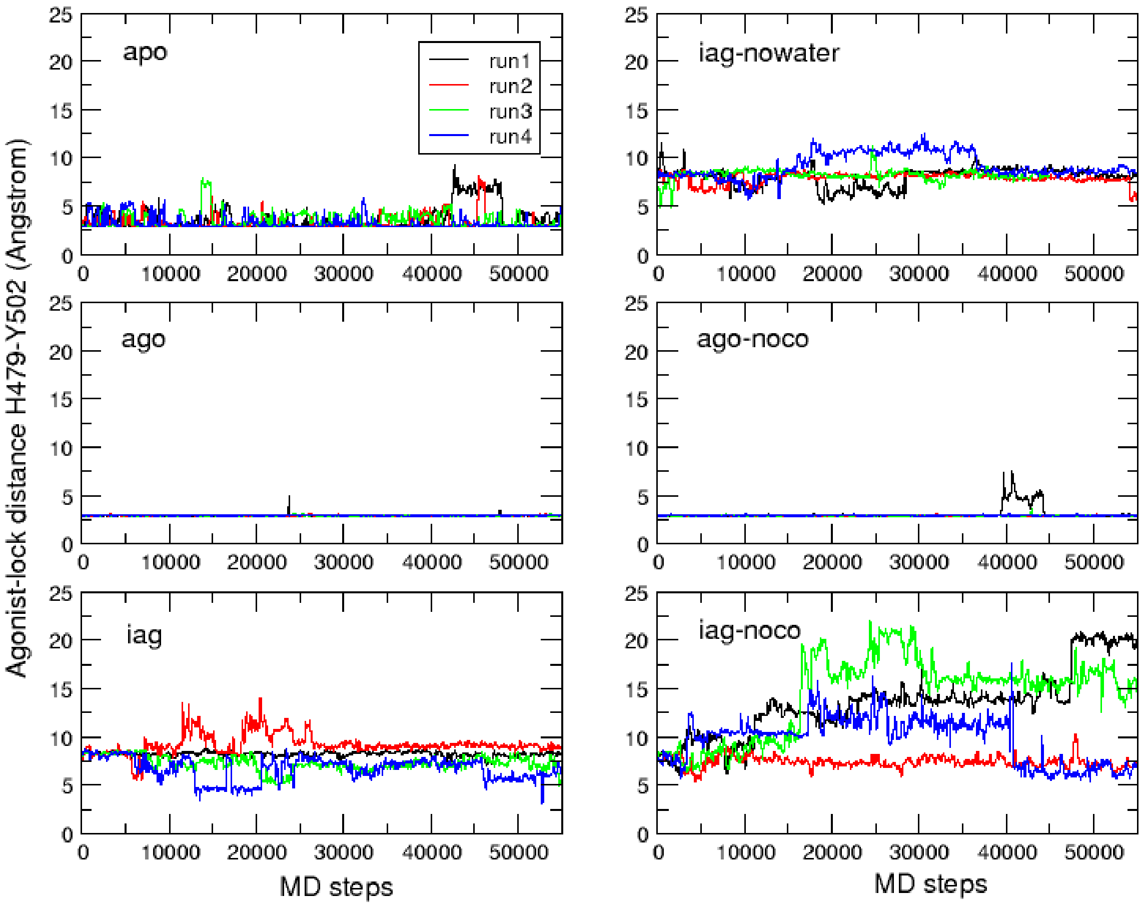

Figure S1. Plot of H479:Nε-Y502:OH distance (Y-axis in Angstroms) averaged over interval of 1ns along the MD trajectories (X-axis as number of steps that divided by 100 for conversion to ns-time scale) for the 6 simulated systems, where two independent trajectories are shown in black and red color.

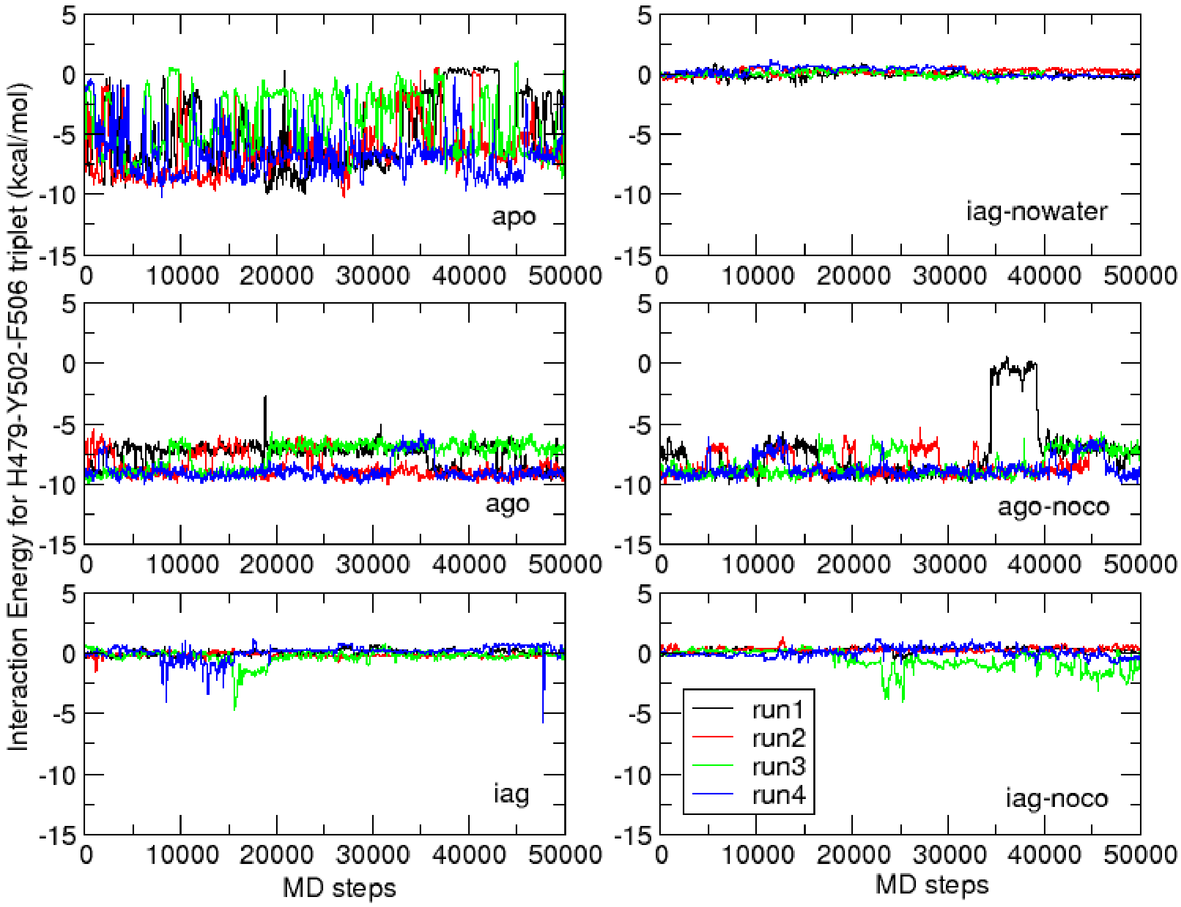

Figure S2. Plot of the interaction energies for the H479-Y502-F506 triplet (Y-axis in kcal/mol) averaged over interval of 1ns along the MD trajectories (X-axis as number of steps that divided by 100 for conversion to ns-time scale) for the 6 simulated systems, where four independent trajectories are shown.

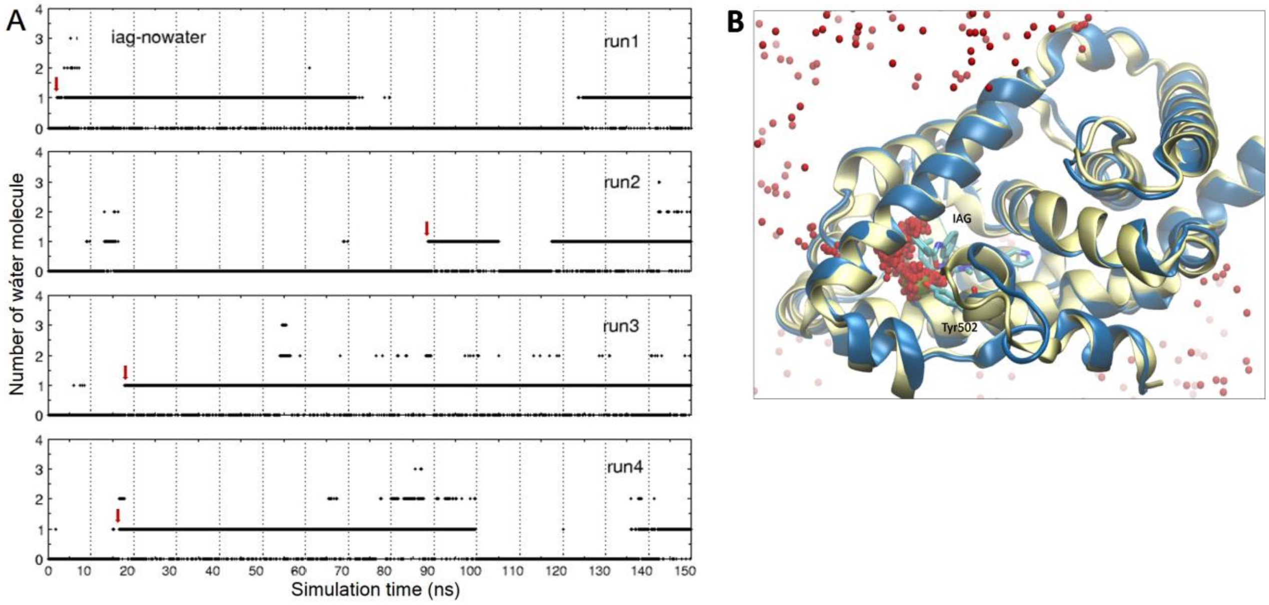

Figure S3. (A) Plot of number of water molecule within 3 Å from Y502:OH atom (the 1st water shell) during the initial 150ns (X-axis is simulation time scale), for four simulations of the inverse agonist **10**-LBD complex starting with and without the single water molecule found in its crystal structure (iag and iag_nowat, respectively). The red arrows indicate when the first water molecule started to diffuse back into the pocket. (B) The diffusion pathway of water from the bulk solvent back into its original position found in crystal structure (protein in yellow color and green sphere represented crystal water).

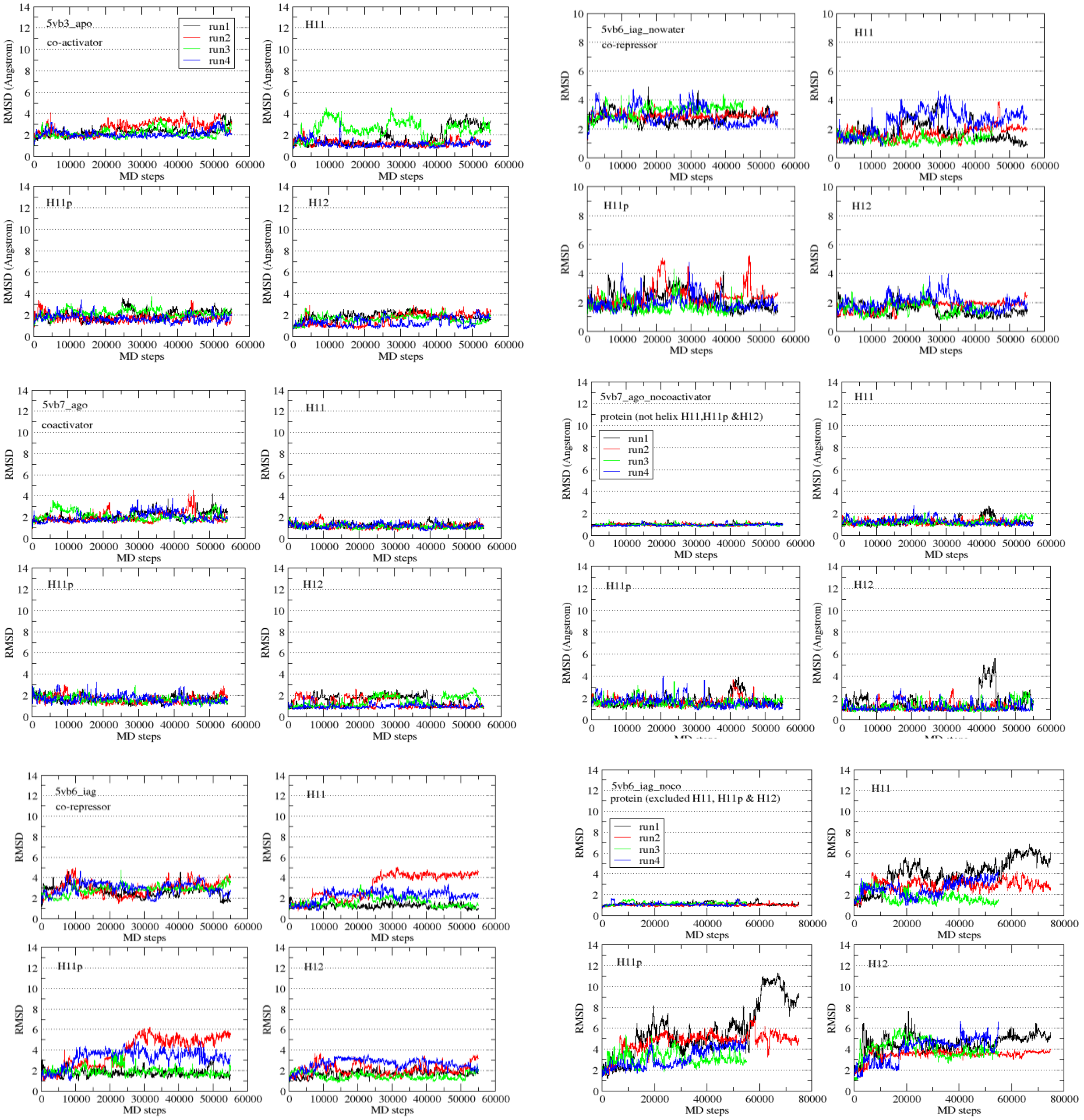

Figure S4. Plot of Root-mean-square-deviation (RMSD) of the backbone atoms of helix H11, H11′, H12 and co-activator (Y-axis in Angstrom) averaged over interval of 1ns along the MD trajectories (X-axis as number of steps that divided by 100 for conversion to ns-time scale) for the 6 simulated systems, where four independent 0.5μs trajectories are shown in black, red, green and blue color.

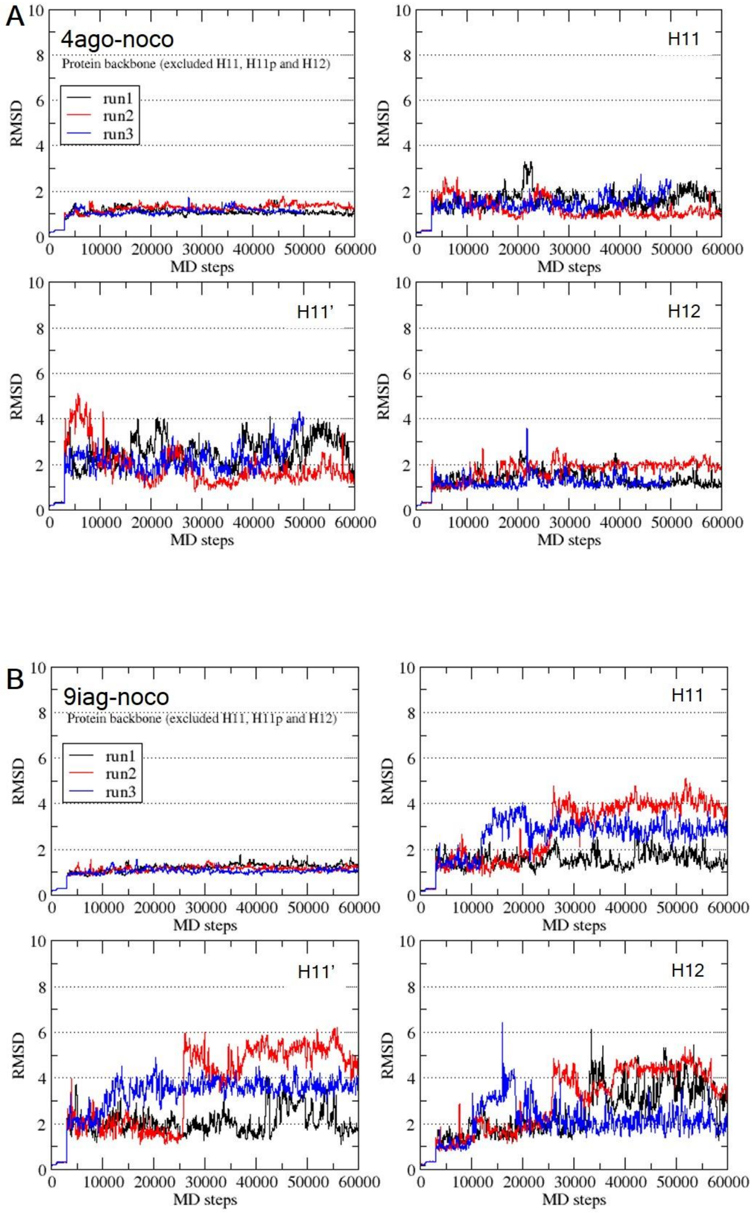

Figure S5. Plot of Root-mean-square-deviation (RMSD) of the backbone atoms of helix H11, H11´, H12 and co-activator (Y-axis in Angstrom) averaged over interval of 1ns along the MD trajectories (X-axis as number of steps that divided by 100 for conversion to ns-time scale) for the Biogen systems; agonist-bound complex (4ago-noco) and inverse agonist bound complex (9iag-noco), where three independent 0.6μs trajectories are shown in black, red and blue color.
